# Elevated Heparin-Induced Antibodies Are More Common in Diabetic Patients with Vascular Disease

**DOI:** 10.1155/2014/649652

**Published:** 2014-02-06

**Authors:** Joseph J. Naoum, Nibal R. Chamoun, Mitul S. Patel, Tiffany K. Street, Mazen Haydar, Jean Bismuth, Hosam F. El-Sayed, Mark G. Davies, Alan B. Lumsden, Eric K. Peden

**Affiliations:** ^1^Lebanese American University and University Medical Center Rizk Hospital, P.O. Box 11-3288, Zahar Street, Achrafieh, Beirut, Lebanon; ^2^Houston Methodist and DeBakey Heart and Vascular Center, Houston, TX, USA

## Abstract

*Background*. Hypercoagulable disorders can lead to deep vein thrombosis (DVT), arterial thrombosis or embolization, and early or recurrent bypass graft failure. The purpose of this study was to identify whether diabetes increased the likelihood of heparin-induced platelet factor 4 antibodies in at risk vascular patients. *Methods*. We reviewed clinical data on 300 consecutive patients. A hypercoagulable workup was performed if patients presented with (1) early bypass/graft thrombosis (<30 days), (2) multiple bypass/graft thrombosis, and (3) a history of DVT, pulmonary embolus (PE), or native vessel thrombosis. Relevant clinical variables were analyzed and compared between patients with diabetes (DM) and without diabetes (nDM). *Results*. 85 patients (47 women; age 53 ± 16 years, range 16–82 years) had one of the defined conditions and underwent a hypercoagulable evaluation. Screening was done in 4.7% of patients with early bypass graft thrombosis, 60% of patients were screened because of multiple bypass or graft thrombosis, and 35.3% had a previous history of DVT, PE, or native vessel thrombosis. Of the 43 patients with DM and 42 nDM evaluated, 59 patients (69%) had an abnormal hypercoagulable profile. An elevated heparin antibody level was present in 30% of DM and 12% of nDM patients (chi-squared test *P* < 0.04). Additionally, DM was associated with a higher likelihood of arterial complications while nDM was associated with a higher rate of venous adverse events (chi-squared test *P* < 0.003). *Conclusions*. Diabetes is associated with a higher likelihood of developing heparin-induced antibodies and an increased combined incidence of arterial complications that include early or multiple bypass/graft thrombosis. This finding may influence the choice of anticoagulation in diabetic patients at risk with vascular disease.

## 1. Introduction

Inherited or acquired hypercoagulable states represent a cause of morbidity in patients with vascular disease. Hypercoagulable disorders can lead to deep vein thrombosis (DVT), arterial thrombosis or embolization, and early or recurrent bypass graft failure [1, 2]. The latter have usually been attributed to a failure of surgical technique rather than a hypercoagulable-mediated event. Patients are usually considered to have a hypercoagulable state when they have a laboratory or a clinical picture associated with an increased thrombotic risk. Patients with diabetes mellitus (DM) are at an increased risk of cardiovascular complications as a result of cerebrovascular, cardiac, or peripheral arterial disease [2]. Diabetic patients exhibit a heightened activation of platelets and clotting factors [3] which is also enhanced by the presence of hypertension (HTN) [4]. Platelet hyperactivity in DM can be demonstrated by the presence of increased circulating platelet aggregates and the presence of higher plasma levels of platelet release products such as platelet factor 4 (PF4) [5]. These findings support the concept that DM can be associated with a hypercoagulable state which can be potentially exacerbated in patients with overt arterial disease or those who have undergone vascular intervention.

Heparin-induced thrombocytopenia (HIT) is an immune mediated reaction that results from the exposure to heparin products. Antibodies against epitopes within PF4 form when this protein interacts with heparin and binds to mucopolysaccharides or glycosaminoglycans. These antibodies bind the heparin-PF4 (HPF4) complex and cause platelet activation [6, 7]. The frequency of clinical thrombocytopenia is variable but has been reported to be between 0.5 and 6.5% [8, 9]. Even with exposure to heparin, there exists a majority of patients who do not develop HIT but have HPF4 antibodies present that has been reported to vary between 13% and 50% [8, 10]. Yet, some healthy individuals who have not been previously exposed to unfractionated heparin may demonstrate antibodies for HPF4 with a frequency of 1.4% [11]. The presence of HPF4 antibodies has been shown to independently increase the risk of adverse cardiovascular events even without the evidence of clinically significant thrombocytopenia [10, 12, 13]. The purpose of this study was to identify whether diabetes increased the likelihood of developing HPF4 antibodies in at risk vascular patients.

## 2. Materials and Methods

### 2.1. Study Design

We retrospectively reviewed clinical data on 300 consecutive patients over the age of 18 years. A hypercoagulable workup was performed if patients presented with (1) early bypass/graft thrombosis (<30 days) using either autologous vein or synthetic graft, (2) multiple bypass/graft thrombosis, and (3) a history of DVT, pulmonary embolus (PE), or native vessel thrombosis. Relevant clinical variables were analyzed and compared for patients with diabetes (DM) and without diabetes (nDM). Study data was obtained via a review of the patient's medical records as part of an institutional review board-approved protocol.

### 2.2. Study Setting

The study was performed at the Houston Methodist Hospital that is an academic medical center with 1000 beds in a catchment area of 5 million people. It is a tertiary and quaternary referral facility.

### 2.3. Technique

Specifically, HPF4 antibody measurements were performed centrally at the Houston Methodist Hospital clinical chemistry laboratory. Total antibodies were measured by enzyme-linked immunosorbent assay (*ELISA*) (GTI Diagnostics, Waukesha, WI) and results were obtained as optical density (OD) and reported as positive when above the reference laboratory standard (OD > 0.4).

### 2.4. Statistics

The chi-squared test was used to compare categorical variables. Calculations were performed using Excel software.

## 3. Results

We reviewed clinical data on 300 consecutive patients. A hypercoagulable workup was performed if patients presented with (1) early bypass/graft thrombosis (<30 days), (2) multiple bypass/graft thrombosis, and (3) a history of DVT, pulmonary embolus (PE), or native vessel thrombosis. Eighty five patients (47 women; age 53 ± 16 years, range 16–82 years) had one of the defined conditions and underwent a hypercoagulable evaluation. Screening was done in 4.7% of patients with early bypass graft thrombosis, 60% of patients were screened because of multiple bypass or graft thrombosis, and 35.3% had a previous history of DVT, pulmonary embolus (PE), or native vessel thrombosis. All DM patients in the group had hypertension and were more likely to have coronary artery disease, peripheral arterial disease, end-stage renal disease (ESRD), and hypercholesterolemia than the nDM group (chi-squared test *P* < 0.05) (Table 1). Of the 43 patients with DM and 42 nDM evaluated, 59 patients (69%) had an abnormal hypercoagulable profile with an elevated HPF4 antibody present in 30% of DM and 12% of nDM (chi-squared test *P* < 0.04) (Table 2). All DM patients were in a stable treated phase and none were newly diagnosed. Briefly, we also found that lupus anticoagulant was present in 35% of DM and 33% of nDM, functional protein S deficiency in 19% and 21%, antithrombin III deficiency in 16% and 14%, functional protein C deficiency in 14% and 7%, and anticardiolipin antibodies in 9% and 7%, respectively. Yet, none of these reached statistical significant difference between the two groups.

Group analysis showed that DM was associated with a higher combined likelihood of arterial complications that included early bypass/graft thrombosis (<30 days) or multiple bypass/graft thrombosis (chi-squared test *P* < 0.003). On the other hand, nDM was associated with a higher combined rate of venous adverse events such as DVT, PE, or superficial vein thrombosis (chi-squared test *P* < 0.003). Additionally, when comparing DM patients with ESRD on hemodialysis (*n* = 31) and DM without ESRD not undergoing dialysis (*n* = 12), we found no difference in the presence of HPF4 antibodies between these two subgroups (chi-square-test *P* = 0.8).

## 4. Discussion

A study by Diaz and colleagues showed no increase in the presence of HPF4 antibodies in healthy DM patients when compared with a healthy control population. As in our study, antibody titers were measured by ELISA and reported as OD readings [8]. In contrast to that report, our findings show that DM patients at risk with vascular disease were significantly more likely to develop HPF4 antibodies than nDM patients. All patients in our series had presented with either early bypass/graft thrombosis (<30 days), multiple bypass/graft thrombosis, or a history of DVT, PE, or native vessel thrombosis. All had been previously exposed to heparin and 64% of the patients were on dialysis receiving scheduled heparin during treatments three times a week. In this at risk population, an elevated HPF4 antibody was present in 30% of DM and 12% of nDM (chi-squared test *P* < 0.04) without evidence of thrombocytopenia. The presence of HPF4 antibodies without clinical evidence of HIT is not uncommon and our series shows a range consistent with previous reports [10, 12, 14].

Patients on hemodialysis receive heparin regularly during their scheduled treatments and many also develop HPF4 antibodies without a clinical manifestation of thrombocytopenia or HIT [9]. Krane and associates investigated the presence of HPF4 antibodies in hemodialysis patients with DM and found that HPF4 antibodies were present in 18.7% of the patients [15]. Zhao and associates found the presence of HPF4 antibodies in maintenance hemodialysis patients to be lower at 5.6% and demonstrated that DM patients were more likely than nDM patients to develop antibodies [16]. In our study, 29% of DM patients with ESRD undergoing hemodialysis had HPF4 antibodies compared to 25% of DM patients without ESRD not undergoing hemodialysis. There was no difference between the two groups. This may suggest that with DM the routine or frequent exposure to heparin is not necessary for the development of HPF4 antibodies, but rather that a nonfrequent exposure may be all that is needed to stimulate antibody formation. Borsey and colleagues showed that PF4 concentration was elevated in both DM with and without retinopathy compared to nDM controls [17]. Additionally, Roy and associates have shown similar results in DM patients [18]. A possible explanation for the risk of HPF4 formation that extends beyond the duration or frequency of heparin exposure in DM is that persistent heparin exposure may not be necessary for antibody formation to occur against the excess PF4 that exists surface bound to mucopolysaccharides or glycosaminoglycans on the platelet surface [13, 19].

Blank and associates support the concept that endothelial cells (EC) derived from different vascular beds have different characteristic features and can respond differently to injurious agents. According to this concept, EC can integrate different extracellular signals and respond differently to the same injurious agent in distinct regions of the vascular system, whether arterial or venous. As such, the authors demonstrated that activation of microvascular EC by HPF4 antibodies was direct, while activation of large vessel EC occurs indirectly and is associated with platelet activation or the release of cytokines [7]. Diabetes is associated with the development of EC dysfunction, enhanced platelet aggregation and activation [20], increased circulating platelet aggregates, and a higher level of platelet release products, such as beta-thromboglobulin, thromboxane B_2_, and PF4 [3, 5]. Additionally, nicotinamide adenine dinucleotide hydrogenase (NADH) levels are increased with DM and can generate reactive oxygen species which have been known to induce activation of endothelial growth factors responsible for vascular dysfunction [21]. NADH has been shown to significantly stimulate the dose-dependent release of IL-6 and modulate the effects of cytokines on peripheral blood cells [22]. In our study, subgroup analysis showed that DM was associated with a higher combined likelihood of arterial complications that included early or multiple bypass/graft thrombosis (chi-squared test *P* < 0.003). Thus, patients with DM may have experienced both direct activation of microvascular EC by HPF4 antibodies and activation of large vessel EC as a result of the enhanced state of platelet aggregation and cytokine activation postulated above in addition to the associated higher likelihood of developing peripheral arterial disease and vascular complications [2].

Cacciapuoti discussed that antiphospholipid syndrome, polycythemia vera, atrial fibrillation, acquired hyperhomocysteinemia, myeloproliferative disease, and DM are the most frequent causes of an acquired hypercoagulable state responsible for DVT [23]. In a report by Adams and colleagues, no significant increased risk of deep venous thrombosis was found in patients with DM compared with patients nDM in a study population who underwent elective total knee arthroplasty [24]. However, in a recent report by Wang and Zhao, the risk of DVT in patients after total knee arthroplasty with DM was 2.76 times the risk in nDM patients using logistic regression modeling [25]. In a study of 2488 venous thromboembolism patients in which 476 had a clinical history of DM, the latter was associated with a significant increase in the risk of recurrent deep vein thrombosis [26]. In contrast to these reports, our study showed that at risk vascular nDM patients were associated with a higher combined rate of venous adverse events such as DVT, PE, or superficial vein thrombosis (chi-squared test *P* < 0.003) when compared to patients with DM. Heit and colleagues studied 1922 patients over a 25-year period and found that DM was also not an independent risk factor for incident venous thromboembolism in the population studied [27].

All DM patients in our study had hypertension and were more likely to have coronary artery disease, peripheral arterial disease, end-stage renal disease (ESRD), and hypercholesterolemia than nDM. The presence of DM was associated with an increased cardiovascular morbidity and has important implications especially when associated with the presence of HPF4 antibodies. Mattioli and colleagues studied 124 consecutive patients with unstable angina who at baseline did not have HPF4 antibodies and were treated with unfractionated heparin. The combined incidence of death, myocardial infarction, recurrent angina, urgent revascularization, and stroke was 66% in patients with antibodies and 44% in patients without antibodies during a 1-year followup [12]. In an observational study, Kress and associates detected the incidence of preoperative HPF4 antibodies and assessed the associated risk of postoperative adverse events in a nonselected group of patients undergoing cardiac surgery. Of those screened, 5.4% had HPF4 antibodies preoperatively. The presence of HPF4 antibodies was associated with a longer hospital length of stay, higher incidence of prolonged mechanical ventilation, acute limb ischemia, and gastrointestinal and renal complications including the need for hemodialysis. The authors showed that HPF4 antibodies before surgical heparin administration was an independent and clinically significant risk factor for postoperative events following cardiac surgery and recommend that a risk profile of patients should include HPF4 antibody status [28]. In addition, patients who had been previously treated with unfractionated heparin as part of cardiac surgery management and had developed HPF4 antibodies were more likely to die or develop myocardial infarction, pulmonary embolism, or stroke than patients who were antibody negative. These patients retained a higher thrombotic event rate up to a 1-year followup [14].

The present report has several limitations. The scope of this retrospective study focused on measuring HPF4 antibodies in patients who had already presented with complications related to their vascular disease. At the time of screening, all patients had been exposed at least once to unfractionated heparin as part of the management of their vascular disease or complications. However, except for the population on hemodialysis who had scheduled weekly exposure to unfractionated heparin during treatment, we did not collect additional data on the timing or frequency of heparin exposure in the remainder of patients not on hemodialysis. This may be relevant for Mattioli and colleagues have shown that within 6 days of heparin exposure 30% of patients can develop HPF4 antibodies [12]. We did not ascertain whether alternative therapies or modes of anticoagulation in these patients may have had an effect on outcome. Stribling and associates propose to treat the presence HPF4 antibodies in high-risk clinical scenarios with nonheparin anticoagulants or direct thrombin inhibitors [13]. However, limited evidence is available to guide on the optimal nonheparin anticoagulant to be used during vascular surgery with suspected or documented HIT [29, 30]. The ELISA immunoassay is a quantitative test capable of detecting HPF4 antibodies [31]. It is a sensitive antigen-based assay with high negative predictive value but a variable specificity between 50% and 89%. This results in the detection of both nonpathogenic and pathogenic antibodies in patients with and without clinical HIT. The utility of this test is ruling out the presence of HPF4 antibodies during the HIT workup. The 14C-Serotonin release assay (SRA) is a functional test to detect antibodies capable of activating platelets which has a specificity and sensitivity of more than 95%. Despite being one of the reference standard tests for the detection of HPF4 antibodies, this test is not readily available in all laboratories [31–33]. We did not utilize the functional SRA tests to determine if the associated thrombotic events were due to immunogenic heparin-induced antibodies capable of activating platelets. We believe that our observations may contribute to the understanding of the relation between DM and HPF4 antibodies in at risk vascular patients. We intend that the data generated in this work be validated by prospective studies in the future.

## 5. Conclusions

Diabetes is associated with a higher likelihood of developing heparin-induced antibodies and an increased combined incidence of arterial complications that include early or multiple bypass/graft thrombosis. The routine or frequent exposure to heparin as observed in ESRD patients on hemodialysis did not increase the risk for HPF4 antibody formation in DM patients. Our findings may help stratify patients at risk for vascular complications and potentially influence the choice of anticoagulation especially in DM patients who are at risk with vascular disease.

## Figures and Tables

**Table 1:** Comparison of patient characteristics for both diabetics (DM) and nondiabetics (nDM). CAD: Coronary artery disease, PVD: peripheral vascular disease, A-fib: atrial fibrillation, COPD: chronic obstructive pulmonary disease, CKD: chronic Kidney Disease, ESRD: end-stage renal disease, Chol: hypercholesterolemia, and AAA: abdominal aortic aneurysm.

Demographics	Number of patients	
	DM	nDM
Men	21	17
Women	22	25
Average age (yrs)	54 ± 13	54 ± 18
CAD	22	9*
Hypertension	43	32*
PVD	16	6*
A-fib	2	8*
COPD	8	8
Varicose veins	0	3
CKD 3-4	2	3
ESRD	32	22*
Chol	20	9*
AAA	0	4*

*Chi-squared *P* < 0.05.

**Table 2:** Patients with diabetes (DM) and without diabetes (nDM) presenting with acquired heparin platelet factor 4 (HPF4) antibodies.

HPF4 antibodies	DM (%)	nDM (%)	Total (%)
Present	13 (30)*	5 (12)	18 (21)
Not present	30 (70)	37 (88)	67 (79)

Total	43 (51)	42 (49)	85 (100)

*Chi-squared test *P* < 0.04.
